# Combination of Haloperidol With UNC9994, β-arrestin-Biased Analog of Aripiprazole, Ameliorates Schizophrenia-Related Phenotypes Induced by NMDAR Deficit in Mice

**DOI:** 10.1093/ijnp/pyae060

**Published:** 2024-11-29

**Authors:** Tatiana V Lipina, Huy Giang, Jonathan S Thacker, William C Wetsel, Marc G Caron, Jean Martin Beaulieu, Ali Salahpour, Amy J Ramsey

**Affiliations:** Department of Pharmacology & Toxicology, University of Toronto, Toronto, Ontario, Canada; Department of Pharmacology & Toxicology, University of Toronto, Toronto, Ontario, Canada; Lunenfeld-Tanenbaum Research Institute at Mount Sinai Hospital, Toronto, Ontario, Canada; Department of Neurobiology, Duke University Medical Center, Durham, North Carolina, USA; Department of Psychiatry and Behavioral Sciences, Duke University Medical Center, Durham, North Carolina, USA; Mouse Behavioral and Neuroendocrine, Analysis Core Facility, Duke University Medical Center, Durham, North Carolina, USA; Department of Cell Biology, Duke University Medical Center, Durham, North Carolina, USA; Department of Neurobiology, Duke University Medical Center, Durham, North Carolina, USA; Department of Psychiatry and Behavioral Sciences, Duke University Medical Center, Durham, North Carolina, USA; Mouse Behavioral and Neuroendocrine, Analysis Core Facility, Duke University Medical Center, Durham, North Carolina, USA; Department of Cell Biology, Duke University Medical Center, Durham, North Carolina, USA; Department of Pharmacology & Toxicology, University of Toronto, Toronto, Ontario, Canada; Department of Pharmacology & Toxicology, University of Toronto, Toronto, Ontario, Canada; Department of Pharmacology & Toxicology, University of Toronto, Toronto, Ontario, Canada

**Keywords:** NMDA, Grin1, MK-801, UNC9994, haloperidol, Akt, GSK-3, CaMKII

## Abstract

**Background:**

Glutamatergic system dysfunction contributes to a full spectrum of schizophrenia-like symptoms, including the cognitive and negative symptoms that are resistant to treatment with antipsychotic drugs (APDs). Aripiprazole, an atypical APD, acts as a dopamine partial agonist, and its combination with haloperidol (a typical APD) has been suggested as a potential strategy to improve schizophrenia. Recently, an analog of aripiprazole, UNC9994, was developed. UNC9994 does not affect dopamine 2 receptor (D2R)-mediated Gi/o protein signaling but acts as a partial agonist for D2R/β-arrestin interactions. Hence, one of our objectives was to probe the behavioral effects of co-administrating haloperidol with UNC9994 in the N-methyl-D-aspartate receptor (NMDAR) mouse models of schizophrenia. The biochemical mechanisms underlying the neurobiological effects of dual haloperidol × UNC9994 action are currently missing. Hence, we aimed to explore D2R- and NMDAR-dependent signaling mechanisms that could underlie the effects of dual drug treatments.

**Methods:**

NMDAR hypofunction was induced pharmacologically by acute injection of MK-801 (NMDAR pore blocker; 0.15 mg/kg) and genetically by knockdown of Grin1 gene expression in mice, which have a 90% reduction in NMDAR levels (Grin1 knockdown [Grin1-KD]). After intraperitoneal injections of vehicle, haloperidol (0.15 mg/kg), UNC9994 (0.25 mg/kg), or their combination, mice were tested in open field, prepulse inhibition (PPI), Y-maze, and Puzzle box. Biochemical effects on the phosphorylation of Akt, glycogen synthase kinase-3 (GSK-3), and CaMKII in the prefrontal cortex (PFC) and striatum of MK-801-treated mice were assessed by western blotting.

**Results:**

Our findings indicate that low dose co-administration of UNC9994 and haloperidol reduces hyperactivity in MK-801-treated animals and in Grin1-KD mice. Furthermore, this dual administration effectively reverses PPI deficits, repetitive/rigid behavior in the Y-maze, and deficient executive function in the Puzzle box in both animal models. Pharmacological inhibition of NMDAR by MK-801 induced the opposite effects in the PFC and striatum on pAkt-S473 and pGSK3β-Ser9. Dual injection of haloperidol with UNC9994 reversed MK-801-induced effects on pAkt-S473 but not on pGSK3β-Ser9 in both brain structures.

**Conclusions:**

The dual administration of haloperidol with UNC9994 at low doses represents a promising approach to ameliorate symptoms of schizophrenia. The combined drug regimen elicits synergistic effects specifically on pAkt-S473, suggesting it as a potential biomarker for antipsychotic actions.

**Significance statement**

Schizophrenia is a devastating mental disorder and characterized by positive, negative, and cognitive symptoms. Research on effective antipsychotic drugs (APDs) focuses on cognitive and negative symptoms. Aripiprazole, an atypical APD, acts as a dopamine partial agonist, and its combination with haloperidol (a typical APD) has been suggested as a potential strategy to improve schizophrenia symptoms. An analog of aripiprazole, UNC9994, does not affect D2R-mediated Gi/o protein signaling but acts as a partial agonist for D2R/β-arrestin interactions. Our findings with pharmacological (MK-801) and genetic (Grin1-KD) mouse models of NMDAR deficiency showed that the dual administration of UNC9994 with haloperidol at low doses reduces hyperactivity, corrects prepulse inhibition (PPI) deficits, rigid behavior in the Y-maze, and deficient executive function in the Puzzle box. The MK-801-induced phosphorylation of Akt at Serine-473 was corrected with co-treatment of haloperidol with UNC9994. Further studies of the polypharmacy of UNC9994 with APDs are essential to facilitate translational studies in clinics.

## INTRODUCTION

Schizophrenia is a devastating mental disorder affecting nearly 1% of the population and accounting for 25% of psychiatric hospital beds. Its symptomatic structure includes positive, negative, and cognitive impairments (Elvevåg and Goldberg, 2000). Cognitive impairments are a core of schizophrenia (Elvevåg and Goldberg, 2000) as they are observed among all subtypes of schizophrenia (Heinrichs et al., 1993).

Cognitive and negative symptoms respond minimally to currently available drugs (Keefe, 2007; Miyamoto et al., 2012) and remain a focus of intense research dedicated to antipsychotic drug (APD) development. Besides APDs, other drugs have been re-purposed in attempts to correct some symptoms of schizophrenia, including, eg, anticholinergic drugs, antidepressants, treatments to manage obesity, type II diabetes, cardiovascular diseases, or sleep disturbances (McCutcheon et al., 2023), but their effectiveness is still unclear. Several conceptually novel treatments were introduced to alleviate some cognitive symptoms of schizophrenia, including, eg, trace amine-associate receptor 1 agonists (Koblan et al., 2020), M1/M4 muscarinic receptor agonist KarXT (Kaul et al., 2024), cannabidiol (McGuire et al., 2018), riluzole and memantine (Farokhnia et al., 2014), glycine transporter inhibitor (Fleischhacker et al., 2021), or luvadaxistat, a d-amino acid oxidase inhibitor (Fradley et al., 2023). Although no pharmacological treatments have received official approval for preventing or treating the cognitive symptoms of schizophrenia, significant advancements in biomedical research may offer hope in addressing these challenges (Lobo et al., 2022).

The difficulty in designing APDs arises from our incomplete understanding of the molecular causative mechanisms of schizophrenia. Genetic studies point toward high polygenicity of schizophrenia suggesting that higher clinical efficacy might be achieved with multitarget drugs rather than single-target compounds (Hopkins, 2008). For instance, our previous study showed a synergistic effect between phosphodiesterase 4B and glycogen synthase kinase-3 (GSK-3) inhibitors to correct schizophrenia-related behavioral endophenotypes in DISC1-L100P mutant mice (Lipina et al., 2011), supporting the idea of multitarget drugs in mental health disorders (Talevi et al., 2012).

All current APDs act via antagonism of the dopamine D2R (Seeman and Lee, 1975; Miyamoto et al., 2012; Kaar et al., 2020). In addition to the cAMP-dependent action, D2R activation also can affect other signaling pathways (Beaulieu et al., 2008), eliciting its effects in a G-protein-dependent and independent manner (Masri et al., 2008). G-protein-dependent action is a classical feature of GPCRs, but the G-protein-independent mechanism of D2R is mediated via β-arrestin (Beaulieu et al., 2005). Pharmacological activation of D2R in mice inhibits Akt kinase with concomitant activation of GSK-3 (Beaulieu et al., 2004). Both typical and atypical APDs enhance the phosphorylation of Akt and GSK-3, inhibiting GSK-3 enzymatic activity (Emamian et al., 2004). Thus, it was hypothesized that targeting β-arrestin signaling might be a promising direction for the development of new APDs (Allen et al., 2011). Indeed, a recent study generated 3 new unique compounds, UNC9975, UNC0006, and UNC9994—D2R agonists that display a bias of β-arrestin signaling over G_i_-coupled pathways signaling (Allen et al., 2011). Antipsychotic-like activity of UNC9994 was observed in vivo, but this effect was absent in β-arrestin-2 deficient mice. A follow-up pre-clinical study found robust antipsychotic-like effects of UNC9975 and UNC9994 on phencyclidine (PCP) pharmacological and Grin1 knockdown (Grin1-KD) genetic models of schizophrenia (Park et al., 2016).

All these unique compounds were generated through a robust diversity-oriented modification of aripiprazole, which acts as a partial agonist of the D2 dopamine receptors. Aripiprazole’s mixed agonist/antagonist action is thought to be responsible for its favorable efficacy without producing extrapyramidal side effects. Aripiprazole also has fewer cardio-metabolic effects and hyperprolactinemia than other atypical APDs (Chrzanowski et al., 2006; Newcomer et al., 2008) and is superior in reducing positive and possibly negative symptoms of schizophrenia with minimal risk of re-hospitalization (Pigott et al., 2003).

Antipsychotic *polypharmacy* (co-prescription of 2 or more APDs) is a common practice for treatment-resistant schizophrenia. A recent study hypothesized that dual administration of aripiprazole with haloperidol could be beneficial for patients with schizophrenia (Crapanzano et al., 2022). The study reasoned that when aripiprazole is added to haloperidol, it should displace haloperidol from D2R binding sites and elicit its partial agonist intrinsic activity (Carboni et al., 2012), and therefore attenuate haloperidol-induced adverse effects and ameliorate negative symptoms (Shim et al., 2007; Mahgoub et al., 2015).

A clinical case report, indeed, demonstrated a beneficial effect of aripiprazole combined with haloperidol (Kuo and Hwu, 2008). Based on this study, it could be suggested that the combination of haloperidol with UNC9994 may also elicit specific and potent antipsychotic-like activity to correct schizophrenia-related phenotypes. Furthermore, although synergy between aripiprazole and haloperidol has been reported, the mechanisms of underlying this synergy remain unclear and raise additional questions to explore. For example, it is possible that the addition of haloperidol to UNC9994 may cause a powerful D2R inhibition and reduce UNC9994’s beneficial effects, affecting the correct dose needed for the synergistic studies. Indeed, it is important to carry out studies with dual administration of haloperidol and UNC9994 to find the optimal dosing, duration of treatment to ensure safety, and facilitate the translation of pre-clinical animal findings to human clinical studies. Furthermore, exploration of underlying biochemical events of such drug × drug interaction will shed a light on the detection of sensitive targets for the synergistic action to facilitate the development of new APD and biomarkers.

Therefore, our current study aimed to probe the behavioral and biochemical effects of the dual administration of haloperidol and UNC9994 on pre-clinical models of schizophrenia of the NMDAR hypofunction. Both genetic (Grin1-KD) and pharmacological (MK-801) mouse models show robust schizophrenia-like behaviors (Mohn et al., 1999; Abdallah et al., 2011; Milenkovic et al., 2014; Islam et al., 2017; Mielnik et al., 2021; Lipina et al., 2022), offering valuable tools for testing our hypothesized novel pharmacological strategy.

According to our hypothesis, haloperidol acts as a D2R antagonist, whereas UNC9994 could elicit D2R partial agonism. As such, we probed the phosphorylation of Akt and GSK-3 as main molecular players of the D2R-signaling pathway (Beaulieu et al., 2004, 2005; Emamian et al., 2004) in addition to the pCaMKII-T286 as part of the NMDAR signaling cascade (Tullis et al., 2023). All biochemical events were assessed across 2 brain regions—the prefrontal cortex (PFC) and striatum, critical neurobiological substrates implicated in the psychopathological mechanisms of schizophrenia and the action of APDs (McCutcheon et al., 2020).

## MATERIALS AND METHODS

### Animals

Knockdown mice for the GluN1 subunit of the NMDA receptor (GluN1 knockdown; Grin1-KD) were generated in the animal facility of the University of Toronto as previously described (Mielnik et al., 2021). In Grin1-KD mice, there is an insertion of a neomycin cassette in the intron 17th of the Grin1 gene, flanked by loxP sites. Grin1^+^/^flneo^ C57Bl/6J congenics and Grin1^+^/^flneo^ 129 × 1/SvlmJ congenic mice were intercrossed to produce experimental mice [(Grin1+/+; wild type; WT); and Grin1KD)] as recommended (Silva et al., 1997) to minimize the confound of homozygous mutations on each parental strain. Grin1-KD mice express only 5%-10% of normal levels of the GluN1 subunit of the NMDAR complex.

Two to 5 animals of same-sex (littermates) of mixed genotypes (WT; Grin1+/−; and Grin1-KD) were housed in standard individually ventilated cages (33.1 cm × 15.9 cm × 13.2 cm) (Tecniplast) under controlled temperature (21° ± 1°C), lighting (lights on: 7:00 AM–7:00 PM), and humidity (50%-60%). The animals were given sterile food (Purina mouse chow) and water with ad libitum access. Mice of different genotypes were reared together to avoid potential mortality among Grin1-KD mice when homozygous mice are housed together. This increased mortality could be due to (1) severe skin lesions due to intense self-grooming and (2) loss of body weight due to reduced food consumption (personal unpublished observations).

All experimental behavioral procedures were performed on independent cohorts of naïve male and female WT and Grin1-KD mice between 12 and 16 weeks of age during the light cycle (9:00 AM–5:00 PM). MK-801 pharmacological studies were performed on adult WT mice of both sexes produced from the breeding of Grin1-KD mice. Biochemical and toxicological experiments were conducted on 12-14-weeks-old C57BL/6J male mice purchased from Charles River Laboratories (Charles River Laboratories International, Inc.). The mice were acclimated in the animal colony for 1-2 weeks prior to the start of experiments. Toxicological effects were assessed using 3 behavioral tests on independent sets of mice, including the Inclined platform, Pinch-induced catalepsy, and Rotarod. Pharmacological treatments were assigned according to the Latin square design (Hinkelmann and Kempthorne, 2009). C57BL/6J male mice were used to optimize animal usage for toxicological experiments (Chapman et al., 2013) and to avoid the influence of sex and genetic background on the phosphorylation of the studied proteins (Santana-Santana et al., 2021).

Experimental mice were transported from the mouse colony room to the experimental room for 30 minutes of habituation before any behavioral procedure. All behavior was assessed by a skilled experimenter blind to genotype and drug administration. All procedures were approved by the University of Toronto Faculty of Medicine and Pharmacy Animal Care Committee in compliance with the Animal Research Act of Ontario and the Guidelines of the Canadian Council on Animal Care.

### Behavioral Tests

#### Open Field

Mice were placed in a Plexiglass chamber (20 × 20 × 45 cm3) for 60 minutes. The total distance traveled was measured in 5-minute bins by infrared beam breaks using Versamax activity monitors (Omnitech Electronics, Columbus, Ohio).

#### Prepulse Inhibition of Acoustic Startle Response

Prepulse inhibition (PPI) of the acoustic startle response (ASR) was measured with SRLAB equipment and software from San Diego Instruments as originally described with some modifications (Dulawa et al., 1997). Background white noise was maintained at 65 dB. PPI procedure had 80 randomized trials and was structured as follows: pulse alone (100 dB above background), prepulse alone (4, 8, or 16dB above background), prepulse plus pulse, and no pulse. Five pulse-alone trials were performed at the start and end of the 80 trials. The onset of the pulse following the prepulse was delayed by 100 ms. The time interval between trials was randomized from 5 seconds to 20 seconds. PPI was measured as a decrease in the amplitude of startle response to a 120 dB acoustic startle pulse, following each prepulse (4, 8, and 16 dB). The percentage of PPI induced by each prepulse intensity was calculated as [1—(startle amplitude on prepulse trial)/(startle amplitude on pulse alone)] × 100%.

#### Y-Maze

The Y-maze task was performed as originally described (Arendash et al., 2001). The maze consists of a 3-arm (labeled as arm “A,” arm “B,” and arm “C”) horizontal maze (40 cm × 8 cm × 15 cm) (Noldus Information Technology, The Netherlands) in which the arms are symmetrically disposed at 120° angles from each other. The maze floor and walls were constructed from white opaque polyvinyl plastic with distinctive geometric shapes on the walls. A mouse was initially placed in the start arm (arm “A”), and the sequence (ie, ABCCAB, etc.) and number of arm entries were recorded for 5 min. Arm entries were defined as all 4 paws entering the arm. Spontaneous alternation refers to visiting all 3 arms in sequence (ie, ABC or CAB but not CBC). The percentage of alterations was defined according to the following equation: % Alteration = [(Number of alterations)/(Total arm entries—2)] × 100. The number of revisits of the previously visited arm was manually scored as the index of repetitive/rigid behavior. The total number of arm entries serves as an indicator of ambulation.

#### Puzzle Box

The Puzzle box test was performed as originally described (Ben Abdallah et al., 2011) with mild modifications (Lipina et al., 2013). Mice were tested in 2 sessions with 3 trials per session with an inter-session interval of 1 hour for a total of 6 trials (T1-T6). The Puzzle box was a brightly lit arena (58 × 28 × 27.5 cm^3^) connected to a darkened goal box14 × 28 × 27.5 cm^3^) by a wall divider. In trial T1, mice used a doorway and underpass to reach the goal box. In trials T2, T3, and T4, the doorway was blocked leaving an open underpass. In trials T5 and T6, the underpass was blocked with corncob bedding. This sequence of trials allowed assessing problem-solving ability (T2 and T5) and learning/short-term memory (T3 and T6), while the repetition after 1 hour provided a measure of long-term memory (T4). Each trial started by placing the mouse in the start zone and ended when the mouse entered the goal zone with all 4 paws or after a total time of 3 minutes. The performance of mice in the Puzzle box was assessed by measuring the latency to enter the goal zone.

#### Inclined Platform

A mouse was placed on a wire platform (1 cm × 1 cm mesh), which was then inclined to 90°. The time it took for a mouse to traverse the entire 24 cm length of the platform was recorded. Mice that fell off the inclined platform were assigned a time of 120 seconds (Xie et al., 2007).

#### Pinch-Induced Catalepsy

Was induced as originally described (Amir et al., 1981). A mouse was firmly pinched by the scruff of the neck with a thumb and a forefinger for 5 seconds. The mouse then was gently placed on parallel wooden bars (diameter 0.5 cm), with its front paws positioned at a 45° angle above the hind paws and elevated 8 cm above the experimental table. The duration of freezing (catalepsy) was recorded for up to 2 minutes per trial. Each animal was tested in 3 trials with 2-minute intervals. The average duration of catalepsy was analyzed.

#### Rotarod

Mice were placed on an accelerating rotarod (Ugo Basile, Italy) and were observed at an initial speed of 4 rpm for 30 seconds. The rod was then gradually accelerated at a rate of 0.2 rpm/second. The latency to fall was recorded with a cutoff time of 3 minutes. Mice were given 3 trials with 10-minute rests between trials. The latency to fall for each mouse was counted by averaging over the 3 trials in each day (Xie et al., 2007).

### Western Blot

Drug-treated C57BL/6J mice were sacrificed by cervical dislocation, and the PFC and striatum were quickly dissected on a chilled surgical block within 1-2 minutes as recommended (Beaulieu, 2016). The drug post-treatment time was kept consistent with the design of the behavioral studies (see more details in the “Drugs” section). The collected tissues were frozen in liquid nitrogen and stored at −80°C until further processing.

#### Protein Preparation

Mouse PFC and striatal tissues were homogenized in the radioimmunoprecipitation assay (RIPA) buffer (50 mM Tris-HCl, pH 7.4, 100 mM NaCl, 1% Nonidet P-40, 0.5% sodium deoxycholate, 0.1% SDS; Sigma), with addition of protease and phosphatase inhibitors: 5 mg/mL pepstatin A (Bioshop, PEP605), 1.5 mg/mL aprotinin (Bioshop, APR600), 0.1 mg/mL benzamidine (Bioshop, BEN601), 100 mM PMSF (Bioshop, PMS123), 2.5 mM Na pyro-phosphate (Bioshop, SPP310), 1 mM β-glycerophosphate (Bioshop, GYP001), 10 mM NaF (Bioshop, SFL001), and 1 mM Na3VO4 (Bioshop SOV664) followed by centrifugation at 10 000 r.p.m. for 15 minutes at 4°C. Protein concentration was determined by Pierce^TM^ BCA Protein Assay Kit (Thermo Scientific, 23225). The supernatants were stored at −20°C until further analysis.

#### Phosphorylation of pAkt-S473/Akt and pCaMKII-T286/CaMKII

Whole homogenates (WHs) were thawed on ice, and a colorimetric protein assay (Bio-Rad, Cat#5000112) was used to determine protein concentration. From previously determined optimization, 20 μg of WH lysate was reduced using 2x Laemmli sample buffer (Bio-Rad, Cat#1610737) for 5 minutes. Proteins were electrophoretically separated on 10% sodium dodecyl sulfate (SDS)-polyacrylamide gels prepared from TGX Stain-Free™ FastCast™ Acrylamide Kits (Bio-Rad, Cat#1610183) and run at 150 V for 90 minutes. Next, Tris-Glycine eXtended (TGX) gels were ultraviolet (UV)-activated using stain-free technology on a ChemiDoc MP imaging system (Bio-Rad, Cat#12003154) and then transferred using 2 cycles of the turbo-TGX gel transfer setting (25 V, 2.5 A, 3 minutes) on the Trans-Blot® Turbo™ Transfer System (Bio-Rad, Cat#1704150). Transfer buffer used for blotting consisted of (in % solution): 20 Trans-Blot Turbo 5 × Transfer Buffer (Bio-Rad, Cat#10026938), 20 ethanol, and 60 ultrapure water. Finally, membranes were blocked using EveryBlot Blocking Buffer (Bio-Rad, Cat#12010020) for 30 minutes at room temperature and co-incubated overnight with anti-phosphoT286-CaMKII (CST, Cat#12716), anti-phosphoS473-Akt (CST, Cat#9271), and their respective total protein anti-CaMKII (CST, Cat#50049), anti-panAKT (CST, Cat#2920) at 4°C. On the following day, membranes were incubated with StarBright700 rabbit (1:2500, Bio-Rad#12004161) and StarBright520 mouse (1:2500, Bio-Rad, Cat#12005866) secondary antibodies containing 0.02% sodium dodecyl sulfate at room temperature for 1 hour. Fluorescence intensity was captured and normalized to total protein-specific levels as previously described (Thacker and Mielke, 2022).

#### Phosphorylation of Akt-T308 and GSK-3α/β-Ser21/9

Brain lysates (30 μg) were mixed with 4X loading dye, 0.5 μL of beta-metacaptoethanol (BME), and ddH_2_O to the final volume of 30 μL. After the short spin, all samples were put in the 37°C water bath for 20 minutes for protein denaturation followed by centrifugation at 16 000 r.p.m. for 1 minute at room temperature to completely denature the protein aggregates. The total protein loaded was 25 μL per sample. Proteins were separated on either Novex 10% Tris-Glycine (Invitrogen, XP00100PK2) using 1X Tris-Glycine Running Buffer or NuPAGE^TM^ Bris-Tris Mini Protein Gel, 4%-12% (Thermo Scientific, NP0323BOX) using 1X MOPS Buffer at 120 V for 2 hours, and then transferred to a hydrated polyvinylidene difluoride (PVDF) membrane (Pall Life Sciences, BSP161) using 1X Tris-Glycine Transfer Buffer at 25 V overnight at 4°C. Total protein loading was assessed using a LI-COR Total Protein Stain (LI-COR, LIC-926-11010). Membranes were blocked with 5% bovine serum albumin (BSA) in 1X Tris-buffered saline (TBS) at room temperature for 2 hours on a rocker and immunostained with primary antibodies at 4°C overnight to probe Akt (CST, #2920; 1:1000); GSK-3α/β (CST, #9332; 1:1000); and for 72 hours to probe pAkt-T308 (CST, #9275; 1:500) and pGSK-3α/β-Ser21/9 (CST, #9331; 1:500). Next, membranes were washed in 1X TBST (10 minutes by 3 times), incubated with anti-rabbit IRdye 680CW fluorescence-labeled secondary antibodies for 1 hour at room temperature, followed by washing with 1X Tris-buffered saline with 0.1% Tween**^®^** 20 (TBST) as described before. The immune complexes were visualized on a high-resolution LI-COR imaging machine (LICOR Odyssey M). Membranes were scanned, and fluorescence intensity was captured and analyzed using Empiria studio software (LI-COR Biotech, LLC). All detected signals were normalized to total protein-specific levels as described before (Lipina et al., 2011).

### Drugs

Haloperidol and MK-801 maleate salt were purchased from Sigma-Aldrich (St. Louis, Missouri). UNC9994 was provided by Dr Jian Jin from the University of North Carolina in Chapel Hill. Haloperidol was dissolved in 0.3% Tween-20 (Bio-Rad) and brought to volume with saline 0.9% NaCl. UNC9994 was dissolved in a solution of 0.8% glacial acetic acid in 15% hydroxypropyl β-cyclodextrin in sterile water. MK-801 was dissolved in saline (0.9% NaCl). All compounds were administered intraperitoneally (i.p.) in a 5 mL/kg volume. WT mice were injected with MK-801 or vehicle and returned to their home cage for 10 minutes. Then, MK-801-treated or vehicle-treated WT mice were given either vehicle, haloperidol, UNC9994 alone, or co-treated with haloperidol and UNC9994, and then either immediately placed for the behavioral testing in the open field, or after 20 minutes to test animals in PPI, Y-maze, or Puzzle box tests. As for Grin1-KD mice, drug-treated animals were immediately placed in the open field. UNC9994, haloperidol, and their combination were administrated to Grin1-KD mice with 30 minutes as a pre-treatment time to probe their effects in PPI, Y-maze, and Puzzle box. Vehicle-treated groups included equal number of mice treated with each solvent: saline, 0.3% Tween-20 in saline, and 0.8% glacial acetic acid in 15% hydroxypropyl β-cyclodextrin in sterile water. Given that there was no statistical difference between each solvent-treated animals across experiments, they have been combined and referred to as the “vehicle” group for further analysis. The doses of MK-801 (0.15 mg/kg), UNC9994 (0.25 mg/kg), and haloperidol (0.15 mg/kg) were determined from pilot experiments and based on other studies (Lipina et al., 2005; Duncan et al., 2006; Park et al., 2016).

### Experimental Design

**Experiments 1-4** measured behavioral effects of haloperidol, UNC9994, and their combination on Grin1-KD/WT mice and on MK-801/vehicle-treated WT animals on: (1) motor activity and habituation assessed in the open field; (2) sensorimotor gaiting measured by the PPI test; (3) working memory in Y-maze; and (4) executive functions in the Puzzle box. **Experiment 5** probed the toxicological effects of haloperidol, UNC9994, and their combination on MK-801-treated C57BL/6J male mice assessed in the inclined platform test, pinch-induced catalepsy, and rotarod tests. **Experiments 6-7** aimed to explore biochemical mechanisms underlying the actions of the studied drugs. The PFC and striatum were collected from drug-treated C57BL/6J mice to probe the levels of phosphorylation and total expressions of Akt, GSK-3, and CaMKII by western blotting. The phosphorylation of Akt at S473 (pAkt-S473) and CaMKII at T286 (pCaMKII-T286), along with their total expression, was assessed in a blind manner at the LTRI facility. The phosphorylation of Akt at T308 (pAkt-T308) and GSK-3α/β at Ser21/9 (pGSK-3α/β-Ser21/9), as well as their total expressions, was probed at the University of Toronto facility.

### Statistics

Statistical analyses were performed according to the recommendations described by Gosselin (2019) using TIBCO software (StatSoft, Dell). The collected data were assessed for normality using the Shapiro–Wilk test and for the presence of outliers and extremes by creating classical box and whisker plots, where the upper box value represents the 75th percentile, the lower box value represents the 25th percentile, and the outlier and extreme coefficients are set to 1.5 and 3, respectively. No outliers or extremes were detected, so all raw data were further analyzed. Given that a 2-way analysis of variance (ANOVA) did not detect a significant sex effect, data for both sexes were combined. Behavioral data were analyzed using 2-way ANOVAs with repeated measures with the appropriate between-subjects and within-subjects factors and Pearson’s correlation analysis. Significant main effects or interactions were followed by Fisher’s least significant difference post hoc comparisons. This method was chosen as a liberal test due to the behavioral variability, while still providing statistical power comparable to the Tukey-HSD test (Pereira et al., 2015). Biochemical data were analyzed using the non-parametric Kruskal–Wallis test for multiple independent comparisons, followed by the Mann–Whitney U-test for paired comparison between desired groups due to the small number of biochemical samples per group. Data were graphed using Prism GraphPad (La Jolla, California) software and presented as the mean with the standard error of the mean.

## RESULTS

### Effects of UNC9994 (0.25 mg/kg), Haloperidol (0.15 mg/kg), and their Co-Administration on Hyperactivity in MK-801 (0.15 mg/kg)-Treated and in Grin1-KD Mice

#### MK-801 Pharmacological Model

**Figure 1.**
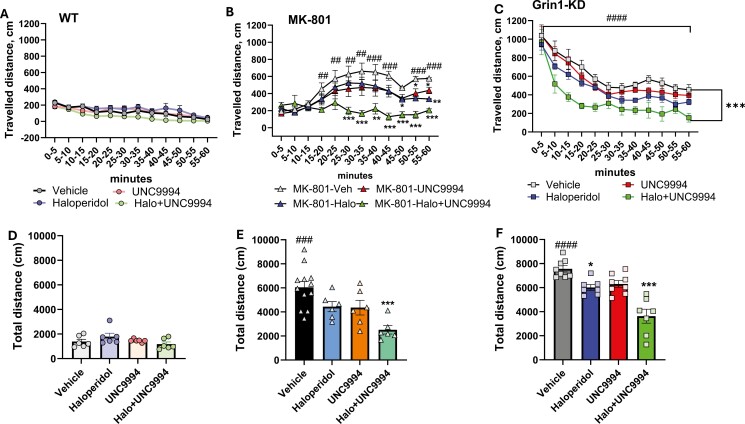
Effects of haloperidol (0.15 mg/kg), UNC9994 (0.25 mg/kg), and their co-administration on hyperactivity assessed in the open field for 60 minutes in vehicle-/WT (A, D), MK-801 (0.15 mg/kg)-treated wild-type (WT) mice (B, E), and Grin1 knockdown (Grin1-KD) mice (C, F). The traveled distance is presented with 5-minutes bin (A-C) and as total ambulation (D-F) for each experimental group: WT (*n* = 6/6/6/6); MK-801-treated mice (*n* = 12/6/6/6) and Grin1-KD (*n* = 8/6/8/6) for vehicle, haloperidol, UNC9994, and Haloperdiol + UNC9994 (Halo + UNC) groups, respectively. *P < .05; **P < .01; *** *P* < .0001 in comparison with MK-801 + vehicle-treated group or vehicle-treated Grin1-KD mice; ##*P* < .01; ###*P* < .001; ####*P* < .0001—in comparison with vehicle-treated WT mice.

The total traveled distance assessed in the open field test was affected by MK-801 treatment [*F*(1, 46) = 78.6; *P* < .0001], co-administered drugs (haloperidol, UNC9994, and haloperidol + UNC9994) [*F*(3, 46) = 6.8; *P* < .0001], and their interactions [*F*(3, 46) = 3.1; *P* < .05]. There was a significant effect of time intervals [*F*(11, 506) = 8.1; *P* < .001], and their interactions either with MK-801 [*F*(11, 506) = 21.3; *P* < .0001], drugs [*F*(33, 506) = 3.3; *P* < .001], or their interactions [*F*(33, 506) = 2.6; *P* < .001] on ambulation. As expected, MK-801 induced hyperactivity (*P*’s < .01-.001) (Figure 1B). Administration of UNC9994 or haloperidol to MK-801-pretreated mice modestly decreased their ambulation (*P*’s < .05-.01), while the dual injection of haloperidol with UNC9994 compound reduced motor activity significantly (*P*’s < .01-.001). At the doses used, haloperidol or UNC9994 administrated alone or in combination did not affect vehicle-treated mice (Figure 1A and D).

#### Grin1-KD Genetic Model

The total traveled distance was significantly affected by genotype [*F*(1, 52) = 406.3; *P* < .0001], drugs [*F*(3, 52) = 11.5; *P* < .0001], genotype × drugs interactions [*F*(3, 52) = 9.2; *P* < .0001], time intervals [*F*(11, 572) = 96.4; *P* < .0001], and time intervals × genotype [*F*(11, 572) = 44.8; *P* < .0001]. Vehicle-treated Grin1-KD mice showed hyperactivity in comparison with vehicle-treated WT littermates in all tested time intervals (*P*’s < .001) (Figure 1C). UNC9994 and haloperidol modestly ameliorated hyperactivity of Grin1-KD mice (*P*’s < .05-.01). Co-administration of UNC9994 with haloperidol substantially reduced their locomotor agitation in the open field (*P*’s < .001).

### Effects of UNC9994 (0.25 mg/kg), Haloperidol (0.15 mg/kg), and their Co-Administration on Sensorimotor Gaiting Deficit in MK-801 (0.15 mg/kg)-Treated and in Grin1-KD Mice

#### MK-801 Pharmacological Model

**Figure 2.**
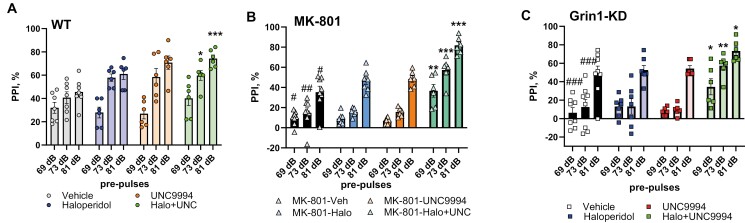
Effects of haloperidol (0.15 mg/kg), UNC9994 (0.25 mg/kg), and their co-administration on sensorimotor gaiting assessed as prepulse inhibition (PPI) of the acoustic startle response in vehicle-/WT (A), MK-801 (0.15 mg/kg)-treated wild-type (WT) mice (B), and Grin1 knockdown (Grin1-KD) mice (C). The percentage of PPI (PPI, %) is presented for each experimental group: WT (*n* = 7/6/6/6); MK-801-treated mice (*n* = 8/7/6/6) and Grin1-KD (*n* = 8/7/6/6) for vehicle, haloperidol, UNC9994, and Haloperdiol + UNC9994 (Halo + UNC) groups, respectively. *P < .05; **P < .01; ***P < .0001 in comparison with MK-801 + vehicle-treated group or vehicle-treated Grin1-KD mice; #*P* < .05; ##*P* < .01; ###*P* < .001 in comparison with vehicle-treated WT mice.

The percentage of PPI was influenced by pre-pulses [*F*(2, 86) = 166.5; *P* < .0001], MK-801 [*F*(1, 43) = 50.5; *P* < .0001], drug treatment [*F*(3, 43) = 16.1; *P* < .0001], MK-801 × drugs interactions [*F*(3, 43) = 8.9; *P* < .0001], pre-pulses × MK-801 [*F*(2, 86) = 12.0; *P* < .0001], and pre-pulses × drugs [*F*(6, 86) = 4.4; *P* < .001] interactions. MK-801-treated mice expressed deficient PPI at all pre-pulses (*P*’s < .05-.01) as compared with vehicle-treated animals (Figure 2A and B). Co-administration of haloperidol with UNC9994 efficiently corrected MK-801-induced PPI impairments (*P*’s < .01-.001).

There was a main effect of MK-801 treatment [*F*(1, 43) = 56.3; *P* < .05] but no effects of drug treatments or their interactions on the ASR intensity (all *P*’s > .05) (Table 1).

**Table 1.** Effects of UNC9994 (0.25 mg/kg), Haloperidol (0.15 mg/kg), and their Combination on the Acoustic Startle Response.

Genotype/pre-treatment	Vehicle	Haloperidol	UNC9994	Haloperidol + UNC9994
Drugs				
**WT/vehicle**	128.6 ± 13.1 (*n* = 7)	201.8 ± 45.7 (*n* = 6)	343.7 ± 82.8^*^ (*n* = 6)	395.8 ± 116.7 (*n* = 6)
**Grin1-KD/vehicle**	1214.8 ± 171.3^###^ (*n* = 8)	1362.9 ± 152.9 (*n* = 7)	1078.1 ± 20.0 (*n* = 6)	929.8 ± 119.3 (*n* = 6)
**WT/MK-801**	225.7 ± 51.5^#^ (*n* = 6)	219.1 ± 43.1 (*n* = 7)	298.0 ± 31.3 (*n* = 6)	232.5 ± 51.2 (*n* = 6)

^#^ *P* < .05.

^###^ *P* < .0001 in comparison with vehicle-treated WT mice.

^*^ *P* < .05 in comparison with vehicle-treated WT mice within the same genotype.

#### Grin1-KD Genetic Model

PPI was substantially affected by pre-pulses [*F*(2, 86) = 95.0; *P* < .0001], genotype [*F*(1, 43) = 29.9; *P* < .0001], drugs [*F*(3, 43) = 6.4; *P* < .001], genotype × drugs [*F*(3, 43) = 5.5; *P* < .01], pre-pulses × genotype [*F*(2, 86) = 12.9; *P* < .0001], pre-pulses × drugs [*F*(6, 86) = 2.7; *P* < .05], and pre-pulses × genotype × drugs [*F*(6, 86) = 2.4; *P* < .05] interactions. The post hoc analysis found that haloperidol co-administered with UNC9994 significantly facilitated PPI at all 3 pre-pulses in Grin1-KD mice (*P*’s < .05-.01) and at 73 dB and 81 dB in WT animals (*P* < .05 and *P* < .001, respectively) (Figure 2A-C).

Measuring the startle response, 2-way ANOVAs detected a main effect of genotype [*F*(1, 43) = 115.2; *P* < .0001] and genotype × drug interactions [*F*(3, 43) = 3.2; *P* < .05]. Vehicle-treated Grin1-KD mice showed enhanced startle (*P* < .001) in comparison to WT mice (Table 1). UNC9994 modestly increased ASR in WT mice (*P* < .05) but there was no effect of haloperidol, UNC9994, or their combination on the ASR in any other experimental groups (*P* > .05).

### Effects of UNC9994 (0.25 mg/kg), Haloperidol (0.15 mg/kg), and their Co-Administration on the Spontaneous Alterations Deficit and Repetitive Behavior Assessed in Y-Maze in MK-801 (0.15 mg/kg)-Treated and in Grin1-KD Mice

#### MK-801 Pharmacological Model

**Figure 3.**
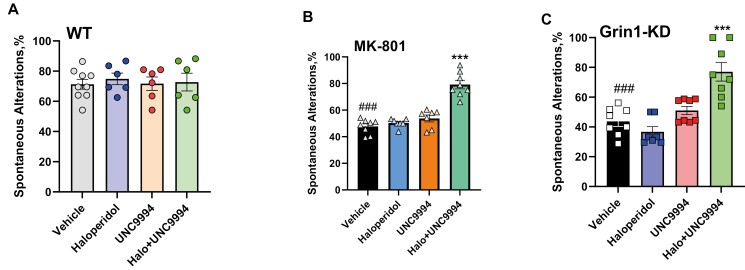
Effects of haloperidol (0.15 mg/kg), UNC9994 (0.25 mg/kg), and their co-administration on the percentage of spontaneous alterations (%) assessed in Y-maze in vehicle-/WT (A), MK-801 (0.15 mg/kg)-treated wild-type (WT) mice (B), and Grin1 knockdown (Grin1-KD) mice (C). WT (*n* = 9/6/6/6); MK-801-treated mice (*n* = 8/7/7/8) and Grin1-KD (*n* = 8/7/8/8) for vehicle, haloperidol, UNC9994, and Haloperdiol + UNC9994 (Halo + UNC) groups, respectively. ***P < .0001 in comparison with MK-801 + vehicle-treated group or vehicle-treated Grin1-KD mice; ###*P* < .001 in comparison with vehicle-treated WT mice.

Assessing the percentage of spontaneous alterations (SA,%), 2-way ANOVA detected a main effect of MK-801 treatment [*F*(1, 47) = 45.8; *P* < .001], drug treatments [*F*(3, 47) = 19.1; *P* < .0001], and MK-801 × drug treatments [*F*(3, 47) = 7.0; *P* < .001]. The post hoc analysis found that co-administration of haloperidol with UNC9994 significantly facilitated working memory in Y-maze (*P* < .001) (Figure 3B), whereas all compounds administered alone did not affect MK-801-treated and vehicle-treated animals (Figure 3A and B). In addition, there was no synergistic effect between haloperidol and UNC9994 on working memory in vehicle-treated mice.

Measuring the number of revisits, 2-way ANOVA found a main effect of MK-801 treatment [*F*(1, 47) = 148.9; *P* < .001], drugs treatment [*F*(3, 47) = 3.1; *P* < .05], and MK-801 × drugs interactions [*F*(3, 47) = 3.2; *P* < .05]. MK-801-treated mice expressed repetitive behavior, revisiting the same arm of the maze significantly more than vehicle-treated animals (*P* < .0001) (Table 2). Dual administration of haloperidol with UNC9994 was able to ameliorate this rigid behavior and reduced the number of revisits (*P* < .0001) in MK-801-treated animals. Haloperidol or UNC9994 given alone had no effect on MK-801 or vehicle-pretreated animals.

**Table 2.** Effects of UNC9994 (0.25 mg/kg), Haloperidol (0.15 mg/kg), and their Combination on the Number of Revisits and the Total Number of Entries in Y-Maze.

Genotype/pre-treatment	Vehicle	Haloperidol	UNC9994	Haloperidol + UNC9994
Drugs				
**Number of revisits**				
** WT/vehicle**	1.6 ± 0.6 (*n* = 9)	1.7 ± 0.7 (*n* = 6)	1.3 ± 0.5 (*n* = 6)	2.2 ± 1.1 (*n* = 6)
** Grin1-KD/vehicle**	15.5 ± 1.5^###^ (*n* = 8)	15.4 ± 1.2 (*n* = 7)	15.1 ± 1.1 (*n* = 8)	6.5 ± 0.9^***^ (*n* = 8)
** WT/MK-801**	12.1 ± 1.4^##^ (*n* = 7)	12.1 ± 1.6 (*n* = 6)	12.7 ± 1.3 (*n* = 6)	6.9 ± 0.8^***^ (*n* = 6)
**Total number of entries**				
** WT/vehicle**	22.9 ± 1.6 (*n* = 9)	19.3 ± 1.3 (*n* = 6)	21.5 ± 2.2 (*n* = 6)	21.0 ± 2.0 (*n* = 6)
** Grin1-KD/vehicle**	49.3 ± 2.2^###^ (*n* = 8)	39.4 ± 2.7^*^ (*n* = 7)	41.5 ± 3.9 (*n* = 8)	21.3 ± 4.3^***^ (*n* = 8)
** WT/MK-801**	37.5 ± 1.5^##^ (*n* = 7)	31.1 ± 3.1^*^ (*n* = 6)	30.3 ± 1.8 (*n* = 6)	22.5 ± 2.7^**^ (*n* = 6)

^##^ *P* < .01.

^###^ *P* < .001 in comparison with vehicle-treated WT mice.

^*^ *P* < .05.

^**^ *P* < .01.

^***^ *P* < .001—in comparison with vehicle-treated Grin1-KD mice or MK-801-treated WT mice.

Measuring the number of entries, 2-way ANOVA detected a main effect of MK-801 [*F*(1, 47) = 23.1; *P* < .01], drug treatments [*F*(3, 47) = 7.4; *P* < .01], and their interactions [*F*(3, 47) = 5.7; *P* < .05]. MK-801-treated mice expressed hyperactivity (*P* < .01), and haloperidol, given alone (*P* < .05) or in combination with UNC9994 (*P* < .01) ameliorated hyperactivity (Table 2).

Pearson’s analysis found a negative correlation between the number of revisits and SA,% (*r* = −0.62; *P* < .001), between the total number of entries and SA,% (*r* = −0.45; *P* < .01), and a positive correlation was found between the number of revisits and total number of visits of Y-maze’ arms (*r* = 0.53; *P* < .01).

#### Grin1-KD Genetic Model

Assessing the SA,%, 2-way ANOVA detected a main effect of genotype [*F*(1,50) = 45.7; *P* < .0001], drugs [*F*(3,50) = 8.0; *P* < .001], and their interactions [*F*(3,50) = 8.5; *P* < .0001]. Vehicle-treated Grin1-KD mice expressed deficient spontaneous alterations compared to WT mice (*P* < .001), which was corrected by the combined administration of haloperidol with UNC9994 (*P* < .001) but not by haloperidol or UNC9994 given alone (Figure 3C).

The number of revisits was significantly affected by genotype [*F*(1, 50) = 245.9; *P* < .001], drugs [*F*(3, 50) = 7.8; *P* < .001], and genotype × drugs [*F*(3, 50) = 10.4; *P* < .0001] interactions. Vehicle-treated Grin1-KD animals expressed profound repetitive behavior, revisiting the same arm of the maze more often than vehicle-treated WT mice (*P* < .0001) (Table 2). Co-administration of haloperidol with UNC9994 reduced the number of revisits in Grin1-KD mice (*P* < .001). Studied compounds given alone had no effects on this parameter in Grin1-KD mice and their WT littermates.

Measuring the total number of entries, 2-way ANOVA detected a main effect of genotype [*F*(1, 50) = 47.8; *P* < .001], drug treatment [*F*(3,50) = 11.5; *P* < .001], and their interactions [*F*(3, 50) = 8.1; *P* < .001]. Vehicle-treated Grin1-KD mice showed hyperactivity (*P* < .001 vs vehicle-treated WT mice), which was slightly reduced by haloperidol (*P* < .05), and a more significant effect was seen after dual co-administration of UNC9994 with haloperidol (*P* < .001) (Table 2).

There was a negative correlation between the SA,% and number of revisits (*r* = −0.76; *P* < .001), as well as between the total number of entries and SA,% (*r* = −0.65; *P* < .001). A positive association was detected between the number of revisits and the total number of visits to Y-maze’s arms (*r* = 0.66; *P* < .001).

### Effects of UNC9994 (0.25 mg/kg), Haloperidol (0.15 mg/kg), and their Co-Administration on the Deficit of the Executive Function Assessed in the Puzzle Box in MK-801 (0.15 mg/kg)-Treated and in Grin1-KD Mice

#### MK-801 Pharmacological Model

Measuring the latency to reach a goal box, 2-way ANOVAs with repeated measures detected a main effect of trials [*F*(5, 205) = 76.8; *P* < .0001], interactions between trials and MK-801 [*F*(5, 205) = 4.9; *P* < .001], trials × co-administrated drugs [*F*(15, 205) = 5.9; *P* < .0001], and trials × MK-801 × co-administrated drugs [*F*(15, 205) = 8.8; *P* < .0001].

**Figure 4.**
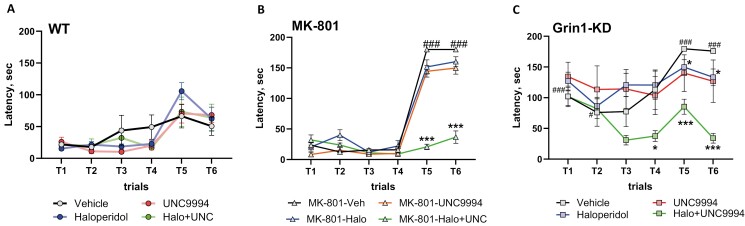
Effects of haloperidol (0.15 mg/kg), UNC9994 (0.25 mg/kg), and their co-administration on executive function, assessed in the Puzzle box paradigm. Latencies scored to reach the goal zone during the 6 trials of the test in vehicle-/WT (A), MK-801 (0.15 mg/kg)-treated wild-type (WT) mice (B), and Grin1 knockdown (Grin1-KD) mice (C). WT (*n* = 6/6/7/6); MK-801-treated mice (*n* = 6/6/6/6) and Grin1-KD (*n* = 6/6/6/6) for vehicle, haloperidol, UNC9994, and Haloperdiol + UNC9994 (Halo + UNC) groups, respectively. *P < .05; ***P < .0001 in comparison with MK-801 + vehicle-treated group or vehicle-treated Grin1-KD mice; ###*P* < .001 in comparison with vehicle-treated WT mice

MK-801-treated mice showed impaired ability to solve the bedding obstacle on trials T5 and T6 (*P*’s < .001) (Figure 4B) in comparison with vehicle-treated animals (Figure 4A). Co-administration of either haloperidol alone or UNC9994 did not affect MK-801-induced impairments on T5 and T6 (*P*’s > .05). Co-administration of haloperidol with UNC9994 markedly facilitated executive functions on T5 and T6 in MK-801-treated animals (*P*’s < .001; Figure 4B).

#### Grin1-KD Genetic Model

The latency to reach a goal box was significantly affected by trials [*F*(5, 200) = 23.9; *P* < .00001], genotype [*F*(1, 40) = 66.5; *P* < .0001], drugs [*F*(3, 40) = 3.4; *P* < .05], genotype × drug interactions [*F*(3, 40) = 3.2; *P* < .05], trials × genotype [*F*(5, 200) = 2.6; *P* < .05], trials × drugs [*F*(15, 200) = 2.1; *P* < .01], and trials × genotype × drugs [*F*(15, 200) = 3.3; *P* < .0001] interactions.

Vehicle-treated Grin1-KD mice expressed deficient performance on T1 (*P* < .001), T2 (*P* < .05), and solving the bedding obstacle on T5 and T6 (*P*’s < .001). Haloperidol given alone was able to modestly improve the performance of Grin1-KD mice on T5 and T6 (*P*’s < .05). Co-administration of haloperidol and UNC9994 significantly improved the performance of Grin1-KD mice in the Puzzle box, facilitating their long-term memory on T4 (*P* < .05) and executive ability to effectively solve bedding obstacles on T5 and T6 (*P* < .001) (Figure 4C). Haloperidol and UNC9994 administered alone or in combination did not affect the behavior of WT mice in the Puzzle box (Figure 4A).

### Effects of UNC9994 (0.25 mg/kg), Haloperidol (0.15 mg/kg), and their Co-Administration on Geotaxis, Catalepsy, Motor Balance, and Coordination Assessed in MK-801 (0.15 mg/kg)-Treated C57BL/6J Male Mice

There were no significant effects of any drug treatments on geotaxis, assessed by the Inclined platform test (all *P*’s > .05), catalepsy measured as the duration of immobility in the Pinch-induced catalepsy (all *P*’s > .05), or motor coordination and motor balance estimated in the Rotarod test (all *P*’s > .05) (Table 3).

**Table 3.** Effects of UNC9994 (0.25 mg/kg) and Haloperidol (0.15 mg/kg) and their Combination on Geotaxis, Catalepsy, and Motor Balance and Coordination in MK-801 (0.15 mg/kg)- and Vehicle-Treated C57BL/6J Male Mice.

	Vehicle				MK-801			
	Vehicle *N* = 8	Haloperidol *N* = 8	UNC9994 *N* = 8	Haloperidol + UNC9994 *N* = 8	Vehicle *N* = 8	Haloperidol *N* = 8	UNC9994 *N* = 8	Haloperidol + UNC9994 *N* = 8
**Inclined platform**								
**Latency, seconds**	17.7 ± 2.2	20.7 ± 5.1	22.7 ± 2.4	27.9 ± 3.3	27.9 ± 7.6	24.2 ± 4.9	23.7 ± 2.5	27.0 ± 2.3
**Catalepsy**								
**Immobility, seconds**	55.1 ± 5.5	52.7 ± 4.5	57.3 ± 4.2	52.3 ± 5.5	48.5 ± 8.5	53.2 ± 2.3	56.7 ± 5.5	49.6 ± 6.1
**Rotarod**								
**Latency, seconds**	100.1 ± 5.8	97.1 ± 3.3	96.1 ± 6.7	90.9 ± 9.3	117.3 ± 16.8	109.3 ± 5.2	95.9 ± 5.2	124.2 ± 27.9

### Effects of UNC9994 (0.25 mg/kg), Haloperidol (0.15 mg/kg), and their Co-administration on the Phosphorylation of Akt (pAkt-S473 and pAkt-T308), GSK3α/β (pGSK3α/β-Ser21/9), and CaMKII (pCaMKII-T286) and their Total Protein Expressions in the *Prefrontal Cortex* of MK-801 (0.15 mg/kg)-Treated C57BL/6J Male Mice

#### pAkt-S473

**Figure 5.**
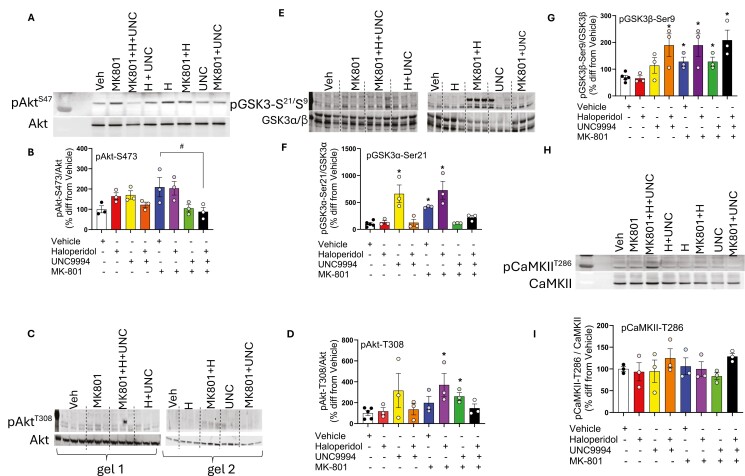
Effects of haloperidol (0.15 mg/kg), UNC9994 (0.25 mg/kg), and their combination on the phosphorylation of Akt (pAkt-S473 and pAkt-T308) (A-D), GSK-3α/β (pGSK-3α/β-Ser21/9) (E-G), and CaMKII (pCaMKII-T286) (H-I) in the prefrontal cortex of MK-801 (0.15 mg/kg)-treated C57BL/6J male mice. Abbreviations: H—haloperidol; MK801, MK-801; MK801 + H, MK-801 + haloperidol; MK801 + H + UNC, MK-801 + haloperidol + UNC9994; MK801 + UNC, MK-801 + UNC9994; UNC, UNC9994, H + UNC, haloperidol + UNC9994; Veh—vehicle. *P < .05 in comparison with vehicle; #*P* < .05 in comparison with MK-801-treated mice; *N* = 3-5 samples per each experimental group.

Kruskal–Wallis revealed a main effect of drug treatments [*H*(7, *n* = 24) = 16.5; *P* < .05] on the level of pAkt-S473 in the PFC. Mann–Whitney U-test detected a trend toward significance, indicating that haloperidol and UNC9994 given alone increased pAkt-S473 level in comparison with the vehicle-treated group (both *P*’s = .08). MK-801 treatment showed a trend toward increasing pAkt-S473 level (*P* = .19). This effect was not significantly altered by either haloperidol or UNC9994 injected alone (both *P*’s > .05). However, the co-administration of haloperidol and UNC9994 significantly reduced the level of pAkt-S473 as compared to the MK-801 group (*P* < .05) (Figure 5A and B).

#### pAkt-T308

Kruskal–Wallis detected a main effect of drug treatments [*H*(7, 26) = 11.8; *P* < .04] on pAkt-T308. Haloperidol and UNC9994 injected alone or in combination did not induce significant effects on pAkt-T308 (*P*’s > .05). MK-801 also had no effect on the phosphorylation of pAkt-T308, but its level was significantly increased after haloperidol or UNC9994 treatments as compared to the vehicle group (both *P*’s < .05) (Figure 5C and D).

All studies with drug treatments did not alter the total expression of Akt in the PFC [*H*(7, 24) = 6.5; *P* > .05, *P* > .05, corresponding to western blots in Figure 5A; and *H*(7, 26) = 9.5; *P* > .05, corresponding to western blots in Figure 5C, respectively] (Supplementary Figure S1A and B).

#### pGSK3α-Ser21

Kruskal–Wallis found a main effect of drug treatments [*H*(7, 26) = 20.3; *P* < .01] on pGSK3α-Ser21 in the PFC. Indeed, the U-test detected an increase of pGSK3α-Ser21 after UNC9994 and MK-801 treatments alone (both *P*’s < .05) (Figure 5E and F). There was no effect of haloperidol given alone, but co-administration of MK-801 with haloperidol increased pGSK3α-Ser21 in the PFC (*P* < .05).

There was a main effect of drug treatments on the level of total expression of GSK3α-Ser21 in the PFC [*H*(7, 26) = 19.9; *P* < .01]. UNC9994 significantly decreased the level of the total expression of GSK3α (*P* < .05), whereas the combination of haloperidol with UNC9994 slightly potentiated the amount of the total GSK3α (*P* = .07) as compared to vehicle. MK-801 significantly reduced the total expression of GSK-3α (*P* < .05), whereas UNC9994 was able to increase the total level of GSK3α after MK-801 treatment (*P* < .05) (Supplementary Figure S1C).

#### pGSK3β-Ser9

There was a significant effect of drug treatments on pGSK3β-Ser9 in the PFC [*H* (7, 26) = 17.5; *P* < .05]. Co-administration of haloperidol with UNC9994 significantly increased pGSK3β-Ser9 as compared to the vehicle group (*P* < .05), whereas no effect was observed once these drugs were given alone (both *P*’s > .05) compared to vehicle. Interestingly, MK-801 also increased pGSK3β-Ser9 (*P* < .05), which was not corrected by haloperidol, UNC9994, or their combination (all *P*’s < .05) (Figure 5E-G).

The total expression of GSK3β was not affected by any drug treatments in the PFC [*H*(7, 26) = 10.7; *P* > .05] (Supplementary Figure S1D).

#### pCaMKII-T286

Kruskal–Wallis test did not detect a main effect of drug treatments on pCaMKII-T286 in the PFC [*H* (7, 24) = 6.2; *P* > .05] (Figure 5H and I).

The total expression of CaMKII in the PFC was not significantly affected by any drug treatments [*H*(7, 24) = 11.6; *P* > .05] (Supplementary Figure S1D). Mann–Whitney U-test found a trend toward significance between vehicle group and haloperidol-, UNC9994-, or MK-801 + UNC9994-treated mice (all *P*’s = .08).

### Effects of UNC9994 (0.25 mg/kg), Haloperidol (0.15 mg/kg), and their Co-Administration on the Phosphorylation of Akt (pAkt-S473 and pAkt-T308), GSK3α/β (pGSK3α/β-Ser21/9), and CaMKII (pCaMKII-T286) and their Total Protein Expressions in the *Striatum* of MK-801 (0.15 mg/kg)-Treated C57BL/6J Male Mice

#### pAkt-S473

**Figure 6.**
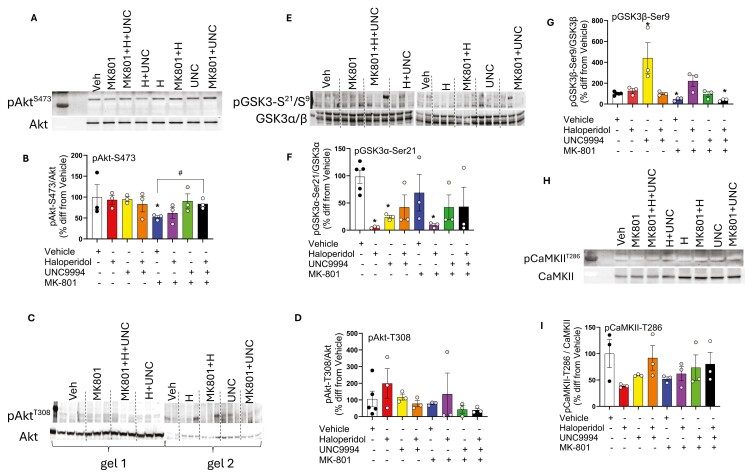
Effects of haloperidol (0.15 mg/kg), UNC9994 (0.25 mg/kg), and their combination on the phosphorylation of Akt (pAkt-S473 and pAkt-T308) (A-D), GSK-3α/β (pGSK-3α/β-Ser21/9) (E-G), and CaMKII (pCaMKII-T286) (H-I) in the striatum of MK-801 (0.15 mg/kg)-treated C57BL/6J male mice. Abbreviations: H, haloperidol; MK801, MK-801; MK801 + H, MK-801 + haloperidol; MK801 + H + UNC, MK-801 + haloperidol + UNC9994; MK801 + UNC, MK-801 + UNC9994; UNC, UNC9994, H + UNC—haloperidol + UNC9994; Veh—vehicle. *P < .05 in comparison with vehicle; #*P* < .05 in comparison with MK-801-treated mice; *N* = 3-5 samples per each experimental group.

Kruskal–Wallis found a main effect of drug treatments on pAkt-S473 in the striatum [*H* (7, 24) = 10.9; *P* ≤ .05]. Indeed, MK-801 reduced the level of pAkt-S473 (*P* < .05), whereas UNC994 given alone (*P* = .08) or in combination with haloperidol (*P* < .05) increased the reduced level of pAkt-S473 induced by MK-801 in comparison with MK-801 group (Figure 6A and B).

#### pAkt-T308

There was no significant effect of drug treatments on pAkt-T308 in the striatum [*H* (7, 26) = 7.5; *P* > .05] (Figure 6C and D).

The total expression of Akt was not affected by drug treatments in the striatum [*H* (7, 24) = 9.1; *P* > .05, corresponding to western blots in Figure 6A; and *H* (7, 26) = 4.5; *P* > .05, corresponding to western blots in Figure 6C, respectively] (Supplementary Figure S2A and B).

#### pGSK3a-Ser21

Kruskal–Wallis found a main effect of drug treatments on pGSK3α-Ser21 in the striatum [*H* (7, 26) = 15.8; *P* < .05]. Haloperidol and UNC9994 reduced pGSK3α-Ser21 (both *P*’s < .05). The haloperidol-induced reduction of pGSK3α-Ser21 in the striatum was also detected after the MK-801 treatment (*P* < .05), but not after UNC9994 or their combination (both *P*’s > .05) in comparison with vehicle or MK-801 groups (Figure 6E and F).

There was a main effect of drug treatment on the level of the total expression of GSK3α in the striatum [*H* (7, 26) = 22.9; *P* < .01]. Haloperidol, UNC9994, and their combination, regardless of MK-801 treatment, increased the total level of GSK3α in the striatum (*P*’s < .05) as compared with the vehicle-treated group (Supplementary Figure S2C).

#### pGSK3β-Ser9

The striking effect of drug treatments was detected on pGSK3β-Ser9 in the striatum [*H* (7, 26) = 19.1; *P* < .01]. UNC9994 given alone increased the level of the pGSK3β-Ser9 (*P* < .05), whereas MK-801 administration significantly reduced the level of the pGSK3β-Ser9 (*P* < .05) in comparison with the vehicle-treated group. U-test found a trend toward significance, indicating that haloperidol can reverse MK-801-induced effect on pGSK3β-Ser9, increasing its level (*P* = .08 in comparison with MK-801 group, *P* = .07 in comparison with vehicle group) (Figure 6E-G). Notably, co-administration of haloperidol with UNC9994 was not efficient to correct MK-801-induced reduction of pGSK3β-Ser9 (*P* < .05) as compared with the vehicle group.

Kruskal–Wallis found a main effect of drug treatments on the total expression of GSK3β in the striatum [*H* (7, 26) = 18.2; *P* ≤ .01]. Co-administration of haloperidol with UNC9994 was able to increase the total level of GSK3β (*P* < .05) in comparison to vehicle-treated mice. Similarly, MK-801 significantly elevated the total level of GSK3β in the striatum (*P* < .05), which had a trend to be normalized by haloperidol (*P* = .08 vs MK-801 group) but was not further modified by UNC9994 or its combination with haloperidol (*P*’s < .05 vs vehicle group) (Supplementary Figure S2D).

#### pCaMKII-T286

There was no significant effect of drug treatments on pCaMKII-T286 level in the striatum [*H* (7, 24) = 10.5; *P* > .05] (Figure 6H and I). However, drug treatments elicited a significant effect on the total level of CaMKII in the striatum [*H* (7, 24) = 15.4; *P* < .05]. There was a trend toward significance for the haloperidol-, UNC9994-, and MK-801 + haloperidol-induced increase of the total CaMKII level in the striatum (all *P*’s = .08) (Supplementary Figure S2E).

## DISCUSSION

Our study revealed the strong efficacy of the combined administration of haloperidol and an analog of aripiprazole, UNC9994, given in low doses, on schizophrenia-related behavioral phenotypes in pharmacological and genetic models of NMDAR hypofunction. The typical APD and UNC9994, given together, elicited beneficial effects on psychomotor agitation and PPI deficit phenotypes related to the positive symptoms of schizophrenia, as well as cognitive phenotypes in both animal models. Haloperidol + UNC9994 demonstrated efficacy on working memory and executive function in an NMDAR-dependent manner but also affected motor functions and sensorimotor gating in vehicle-pre-treated animals, regardless of MK-801 treatment or Grin1 genetic deficiency. Pharmacological inhibition of NMDAR by MK-801 produced contrasting effects on pAkt-S473 and pGSK3-β-Ser9 in the cortex and striatum. Co-administration of haloperidol and UNC9994 reversed MK-801-induced changes in pAkt-S473, but not in pGSK3-β-Ser9, in both brain regions.

### Effects of UNC9994, Haloperidol, and their Combination on Hyperactivity Induced by NMDAR Deficiency

Hyperactivity is a robust phenotype of Grin1-KD mice (Mohn et al., 1999; Duncan et al., 2006; Park et al., 2016; Mielnik et al., 2021) and MK-801-treated animals (Jentsch et al., 1999; Gunduz-Bruce et al., 2009; Mabunga et al., 2019). Construct validity for hyperactivity induced by the NMDAR hypofunction stems from the effect of dissociative anesthetics, such as ketamine and PCP, to induce hallucinations with similarities to the positive symptoms of schizophrenia (Moghaddam et al., 2003; Tamminga et al., 2003). In rats and mice, moderate doses of ketamine and PCP induce locomotor hyperactivity, but at higher doses, lead to ataxia and stereotyped behavior, reducing locomotion (Wu et al., 2005; Mabunga et al., 2019). In our experiment, low doses of MK-801 (0.15 mg/kg) induced hyperactivity in WT mice, gradually increasing the traveled distance after 15 minutes, reaching the maximum level between 30 and 40 minutes, and for the rest of the testing period, supporting previous studies (Mabunga et al., 2019). Genetic deficit of the Grin1 gene that reduces expression of the GluN1 subunit of NMDAR by ~90% (Mohn et al., 1999) also leads to persistent hyperactivity (Milenkovic et al., 2014; Mielnik et al., 2021).

UNC9994 and haloperidol given alone in low doses had a modest effect on locomotor hyperactivity in both models, MK-801-treated animals, and Grin1-KD mice, supporting previous studies (Duncan et al., 2006; Park et al., 2016). Co-administration of the low dose UNC9994 with haloperidol reduced psychomotor to a larger degree in both models without showing any effect on WT animals. While Grin1-KD mice have been used to study APDs, including UNC9994 (Mohn et al., 1999; Duncan et al., 2006; Boulay et al., 2010; Park et al., 2016), to our best knowledge, this current study is the first to assess the effects of the combination of UNC9994 with haloperidol on Grin1-KD mice and the MK-801 pharmacological model. Interestingly, a recent study on rats (Hereta et al., 2019) demonstrated synergetic effects between aripiprazole and antidepressants given in subthreshold doses on the social deficit induced by MK-801 (0.1 mg/kg), as the phenotype related to negative symptoms of schizophrenia (van der Gaag et al., 2006; Wallwork et al., 2012), which is in agreement with other clinical and pre-clinical research (Cho et al., 2011; Kaminska et al., 2015).

A few studies have reported that atypical but not typical APDs can reverse the behavioral effects of NMDAR antagonists. For example, dopamine agonist-induced PPI deficit was reversed by both typical and atypical neuroleptics (Swerdlow et al., 1994); however, atypical antipsychotics rather than typical APDs were more efficient to ameliorate disrupted PPI by the NMDAR-antagonist (Yamada et al., 1999). Another study showed that olanzapine but not haloperidol reduced hyperactivity in the Grin1-KD mice at a dose that did not affect ambulation of the WT mice (Duncan et al., 2006), further supporting the notion about the distinction in efficacy between typical and atypical antipsychotics. Therefore, the achieved synergistic effect between haloperidol and UNC9994 combined at low doses suggests that their co-administration might elicit its effect via signaling pathways that are similar to atypical APDs.

### Effects of UNC9994, Haloperidol, and their Combination on Cognitive Phenotypes Induced by NMDAR Deficiency

Haloperidol and UNC9994 given alone at the studied doses did not affect cognitive behaviors, whereas their dual administration induced robust cognitive facilitation in PPI, working memory, and executive function.

#### Sensorimotor Gating

PPI is a well-established endophenotype of schizophrenia with good face, predictive, and construct validity. Deficits in PPI are reported in schizophrenia (Swerdlow et al., 1994; Braff et al., 2001) and in rodents, PPI is disrupted by NMDAR antagonists (Geyer et al., 2001), including low doses of MK-801 (Lipina et al., 2005). Atypical APDs are more efficacious than typical APDs for correcting the PPI deficit induced by NMDAR hypofunction (Yamada et al., 1999). We found that co-administration of haloperidol with UNC9994 at low doses significantly ameliorated PPI deficit at all 3 pre-pulses in both models without affecting the overall startle response, again suggesting that the co-administration of low-dose haloperidol with UNC9994 might act similar to atypical-like APDs.

#### Working Memory and Executive Functions

Human executive functions can be generally described as a complex of different high-level cognitive processes that enable individuals to regulate their thoughts and actions during a goal–directed behavior by influencing lower level processes (Friedman and Miyake, 2017). These functions include planning, working memory processes, switching between tasks or response inhibition, and their general role is to effectively implement goal–directed actions as well as to control attentional resources (Yogev-Seligmann et al., 2008). Schizophrenia is associated with deficits in executive functions, including impairments of working memory or behavioral flexibility (Wobrock et al., 2009).

#### Y-Maze

In rodents, spontaneous alternation behavior measured in Y-maze requires attention (Katz and Schmaltz, 1980) and working spatial memory. Normally, mice investigate a different arm of the maze rather than revisiting a previously explored one, indicative of sustained cognition. Although deficient spontaneous alterations have been already reported for Grin1-KD mice (Milenkovic et al., 2014; Chen et al., 2018) and MK-801-treated mice (Mabunga et al., 2019), a more detailed behavioral analysis in Y-maze was still missing in these 2 models. We found that both models showed severe cognitive rigidity and more often re-visited the same arms in Y-maze than control animals. Moreover, there was a negative correlation between the SA,% and the number of revisits, highlighting the repetitive behavior as a major reason for the impaired performance of Grin1-KD and MK-801-treated mice in Y-maze. These findings are in good agreement with a recent study (Cleal et al., 2021), where repetitive visits of the previously visited arm were detected as a robust index of cognitive flexibility in Y-maze across a range of experimental conditions and multiple species, including zebrafish, mice, *Drosophila*, and humans. Our results show that a combination of haloperidol with UNC9994 at low doses was able to ameliorate the impaired behavioral performance in Y-maze in both studied models correcting the number of revisits highlighting the combination of haloperidol with UNC9994 as a new approach to correct rigid behavior.

#### Puzzle Box

The Puzzle box was recommended as a relevant and faster way to assess executive functions in a mouse (Ben Abdallah et al., 2011) as compared to the attentional set-shifting (Garner et al., 2006) or the 5-choice serial reaction time (Robbins, 2002), which require long training. It has been shown that Grin1-KD mice have impairments in this paradigm (Milenkovic et al., 2014; Islam et al., 2017; Mielnik et al., 2021), where altered somatosensory functions play an important role in affecting Puzzle box performance in these animals (Lipina et al., 2022). Our current experiments confirmed the Puzzle box impairments in Grin1-KD animals and haloperidol and UNC9994 given alone were not able to correct this deficit. As with the other observations, co-administration of both compounds significantly ameliorated the ability to solve the bedding obstacle on trial 5 and trial 6 and facilitated a long-term memory on trial 4. Importantly, we demonstrated, for the first time, that acute administration of MK-801 severely affected the ability of the experimental mice to solve the bedding problem on trial 5 and trial 6 of the Puzzle box, similar to Grin1-KD mice. Again, this impairment observed in MK-801-treated animals was reversed by the dual pharmacological treatment with haloperidol and UNC9994. The deficits we have observed in the Puzzle box paradigm in both of our models, Grin1-KD and MK-801-treated animals, denote the importance of intact NMDAR signaling on executive function for this assay. In fact, the “bedding” obstacle reflects a highly complex cognitive process, as animals need to extrapolate and find the underpass into a safe, goal box when this route becomes hidden (Abdallah et al., 2011; Poletaeva et al., 2016). In opposite to Grin1-KD mice, MK-801-treated animals performed normally on T1-T4 trials, excluding potential side effects of MK-801 on motor functions or anxiety. This discrepancy between pharmacological and genetic models of NMDAR hypofunction likely reflects the consequences of Grin1 hypofunction on neurodevelopment affecting multiple functions in Grin1-KD mice including hyperactivity, sensory sensitivity, stereotypic, and mood behavior, which could influence behavioral performance of Grin1-KD mice in the Puzzle box (Lipina et al., 2022).

### Polypharmacy Between UNC9994 and Haloperidol: Mechanisms, Limitations, and Future Directions

#### Mechanisms

UNC9994 was discovered as a unique β-arrestin-biased functionally specific to the D2R compound (Allen et al., 2011). It acts as a partial agonist that stimulates D2R-mediated β-arrestin recruitment and signaling, and at the same time, it is inactive at the Gi-dependent pathway. Despite UNC9994 being an agonist for β-arrestin-2 recruitment, in contrast to quinpirole, it does not trigger robust D2R internalization (Allen et al., 2011). The reduced internalization is beneficial, as it may prevent side effects such as tachyphylaxis and receptor downregulation (Allen and Roth, 2011). Another unique feature of the UNC9994 agent is that it is more efficacious (E_max_ = 91 ± 3%) than aripiprazole (E_max_ = 73 ± 1%) to act as a partial agonist for β-arrestin-2 recruitment to D2R (Allen et al., 2011). Haloperidol does not activate D2R-mediated β-arrestin-2 translocation (Granger and Albu, 2005). The addition of haloperidol to UNC9994 may strongly inhibit D2R and subsequently reduce locomotor hyperactivity. From a pharmacological perspective, when UNC9994 is added to haloperidol, 3 substances compete to bind dopamine D2R: UNC9994, a partial agonist of D2R (Ki = 0.75 nM; with slow dissociation koff = 31 min), haloperidol (Ki = 0.57 nM; koff = 1 min), and dopamine (Ki = 16 nM; koff = 24 min) (Pigott et al., 2003; Allen et al., 2011; Sánchez-Soto et al., 2016; de Witte et al., 2018). Hence, when UNC9994 is added to haloperidol, it could replace haloperidol from D2R sites and exert its partial agonist intrinsic activity. As a result, co-administration of UNC9994 with haloperidol could ameliorate negative symptoms and facilitate cognitive deficits as detected in our pre-clinical NMDAR hypofunctional models. Indeed, it has been shown that compounds with partial D2R agonistic activity induced cognitive enhancement in various pre-clinical studies (Kolaczkowski et al., 2015; Thompson et al., 2016). However, future pharmacokinetic studies are needed to assess the absorptions, distributions, metabolism, and excretion (ADME) of haloperidol, UNC9994, given alone and in combination. These studies would determine optimal doses and treatment regimens (sub-chronic, chronic), unlock mechanisms of drug × drug interactions, which will facilitate the development of polypharmacology as a new approach in drug development, with translation to human clinical studies. Nevertheless, our in vivo toxicological findings did not detect any major side effects of the dual administration of haloperidol and UNC9994 in low doses on motor balance, motor coordination, and catalepsy assessed on C57BL/6J mice, excluding potential toxicity of the combined treatments.

Importantly, the balanced functionality of the NMDAR is principally required in the cortex to regulate executive functions and cognitive flexibility. The complex interactions between glutamatergic and dopaminergic systems critically modulate the gating of information flow in a brain, and their impaired interactions lead to several psychopathologies, including schizophrenia (West et al., 2003).

Pharmacological inhibition of NMDAR by noncompetitive antagonist MK-801 affects not only glutamatergic transmission but also dopaminergic release (Tsukada et al., 2005; Bartsch et al., 2015; Saoud et al., 2021), indirectly activating the dopaminergic functions in humans (Kegeles et al., 2000) and rats (Adams et al., 1998). Genetic knockdown of the Grin1 gene significantly affects the dopaminergic functions (Ferris et al., 2014), leading to desensitization of the D2R, a faster spontaneous firing rate of the dopaminergic neurons, accompanied by the attenuated dopamine synthesis and release with increased dopamine clearance.

Currently, the dominant hypothesis of schizophrenia is based on excessive dopaminergic neurotransmission associated with the upregulation of striatal D2 receptors, while cortical hypofunction is linked to NMDAR deficiency (reviewed in McCutcheon et al., 2020). Our biochemical results revealed that acute administration of MK-801 elicited opposite effects on pAkt-S473 and pGSK-3β-Ser9 in the PFC and striatum. MK-801 increased Akt activity while reducing GSK-3β enzymatic activity in the cortex, as evidenced by the increased phosphorylation of Akt at Serine-473 and GSK-3β at Serine9 sites. Conversely, pharmacological inhibition of NMDAR reduced Akt activity in the striatum, coupled with the activation of GSK-3β, as detected by the reduced levels of pAkt-S473 and pGSK-3β-Ser9 in this brain area. Altogether, these data suggest that MK-801 administration reduced D2R activity in the PFC while activating D2R functionality in the striatum, based on the pharmacological effects of D2R agonists and antagonists on Akt and GSK-3 phosphorylation (Beaulieu et al., 2004, 2005).

Consistent with the action of APDs (Emamian et al., 2004), UNC9994 given alone or in combination with a low dose of haloperidol was able to block GSK-3β activity in the striatum and in the PFC, respectively. On the behavioral level, though, these pharmacological treatments were not able to elicit APD-like behavioral effects on control animals, which require modified protocols to capture cognitive enhancement.

Notably, the synergistic behavioral effects between haloperidol and UNC9994 were consistently detected under NMDAR hypofunction conditions, rescuing hyperactivity and cognitive impairments in Grin1-KD mice and MK-801-treated animals, suggesting NMDAR-dependent underlying biochemical mechanisms. Our findings revealed that pAkt-S473 is a sensitive target in response to the combined administration of haloperidol with UNC9994 under MK-801 conditions, consistently detected in both brain areas: PFC and striatum. In other words, the MK-801-induced increase of pAkt-S473 in the cortex was not corrected by haloperidol and UNC9994 given alone but was more effectively reversed by the dual co-administration of the studied drugs. The same pattern was observed in the striatum, although in the opposite direction: the reduced pAkt-S473 level induced by MK-801 was significantly increased by dual injection of haloperidol and UNC9994. These potentiated signaling effects, induced by subthreshold doses of haloperidol and UNC9994, are coherent with behavioral findings, suggesting pAkt-S473 as the best “hit-target” for polypharmacological action. Notably, another phosphorylation site of Akt at Threonine-308 was not responsive to the combined treatment of haloperidol and UNC9994, signifying a specific effect on pAkt-S473. Moreover, since mammalian target of rapamycin complex 2 (mTORC2) regulates phosphorylation of Akt at Serine-473 but not at Threonine-308 (Manning and Toker, 2017), and a recent study detected a role of the NMDAR-Arc-PI3K-mTORC2-Akt complex in synaptic plasticity (Yang et al., 2023), our findings provide new insights for APDs action and drug development.

#### Limitations

Our study, in an attempt to detect the biochemical mechanisms underlying haloperidol × UNC9994 interactions, has several limitations. First of all, the selection of proteins (Akt, GSK-3, CaMKII) to probe their drug-induced phosphorylation is biased, as it is based on already known mechanisms of haloperidol, UNC9994, and MK-801 actions (Seeman and Lee, 1975; Emamian et al., 2004; Beaulieu et al., 2005, 2008; Allen et al., 2011; Miyamoto et al., 2012; Kaar et al., 2020; Tullis et al., 2023). Second, only the MK-801 pharmacological model of the NMDAR hypofunction was used in our current study, with a minimal number of samples per pharmacological group. Moreover, the phosphorylation of the studied proteins was probed only at one-time interval to align with our behavioral findings. However, drug-induced phosphorylation is a dynamic event by nature, as shown, for example, in amphetamine-induced pAkt after 30-, 60-, 90-, and 120-minute post-injection (Beaulieu et al., 2005). Another limitation of our study is that only 2 brain areas were studied: the PFC and striatum, which are major brain targets for dopamine- and glutamate-dependent pathophysiological mechanisms of schizophrenia (McCutcheon et al., 2020). Hence, our biochemical findings can be considered preliminary and require further studies on other pharmacological and genetic models of schizophrenia as the next step towards drug development.

#### Future Directions

A more precise characterization of UNC9994’s action, both alone and in combination with haloperidol and other APDs, is needed, including pharmacokinetics (ADME) as discussed above. Our pre-clinical findings demonstrated the potential to correct cognitive phenotypes related to schizophrenia via dual pharmacological treatments that elicit both antagonism and agonism on the D2R simultaneously. Recently, a new approach was suggested to design and optimize multitarget APDs using an automated system comprising a deep recurrent neural network and a multitask deep neural network (Tan et al., 2020). This system generated new molecular structures with desired polypharmacological profiles, which elicited APD-like effects and were successfully validated using in vivo mouse models. Hence, technological advances to optimize and generate multitarget compounds (Tan et al., 2020), coupled with high-resolution analysis (Khodosevich et al., 2024), will improve further APDs discovery, ultimately enhancing the quality of human lives.

## CONCLUSIONS

Taken together, our pre-clinical experiments show that dual administration of haloperidol and UNC9994 in low doses ameliorates behavioral phenotypes related to positive and cognitive symptoms of schizophrenia on NMDAR hypofunction models, supporting polypharmacy as a new effective approach for the treatment of schizophrenia and development of new APDs. The synergistic effect between haloperidol and UNC9993 was coupled with changes in the phosphorylation of Akt at Serine-473 in the PFC and striatum in the MK-801 pharmacological model. Further validation of the efficacy of the combined treatment to prevent schizophrenia-related behavioral phenotypes in additional genetic mouse models, and elucidation of the precise underlying molecular mechanisms, is needed to generate optimal strategies for translational studies in humans.

## Supplementary Material

pyae060_suppl_Supplementary_Figures

## Data Availability

Detailed statistical analysis and raw data can be obtained upon request to the authors.

## Interest Statement

The authors declare no financial or non-financial interests that are directly or indirectly related to the work submitted for publication. The funders had no role in the design of the study; in the collection, analyses, or interpretation of data; in the writing of the manuscript; or in the decision to publish the results.
