# Supplementary material for: Combination of Haloperidol With UNC9994, β-arrestin-Biased Analog of Aripiprazole, Ameliorates Schizophrenia-Related Phenotypes Induced by NMDAR Deficit in Mice
Source: Int J Neuropsychopharmacol. 2024 Nov 29;27(12):pyae060. doi: 10.1093/ijnp/pyae060 (PMC11656026; doi:10.1093/ijnp/pyae060)
Supplement: pyae060_suppl_Supplementary_Figures [file pyae060_suppl_supplementary_figures.docx]

**Supplementary Information**

**Combination of Haloperidol with UNC9994, β-arrestin-biased analog of Aripiprazole, ameliorates schizophrenia-related phenotypes induced by NMDAR deficit in mice.**

Tatiana V. Lipina^1*^, Huy Giang^1^, Jonathan S. Thacker^2^, William C Wetsel^3-6^, Marc G. Caron^7#^, Jean-Martin Beaulieu^1^, Ali Salahpour^1^, Amy J. Ramsey^1^

**B**

**A**

**Figure S1A-E**: The total expression of Akt (A-B), GSK3-α (C), GSK3-β (D) and CaMKII (E) in the prefrontal cortex of C57BL/6J mice 30 minutes after treatment with vehicle (Veh), haloperidol (H; 0.15 mg/kg), UNC9994 (UNC; 0.25 mg/kg), haloperidol and UNC9994 (H+UNC), MK-801 (0.15 mg/kg), MK-801 and haloperidol (MK801+H), MK-801 and UNC9994 (MK801+UNC), or MK-801, haloperidol and UNC9994 (MK801+H+UNC). The total expression of Akt (A) corresponds to western blot on Figure 5A; the total expression of Akt (B) corresponds to western blot on Figure 5C. * p < 0.05 in comparison with vehicle-treated animals; N = 3-5 samples per each experimental group.

**E**

**C**

**D**

**B**

**A**

**E**

**D**

**C**

**Figure S2A-E**: The total expression of Akt (A-B), GSK3-α (C), GSK3-β (D) and CaMKII (E) in the striatum of C57BL/6J mice 30 minutes after treatment with vehicle (Veh), haloperidol (H; 0.15 mg/kg), UNC9994 (UNC; 0.25 mg/kg), haloperidol and UNC9994 (H+UNC), MK-801 (MK801; 0.15 mg/kg), MK-801 and haloperidol (MK801+H), MK-801 and UNC9994 (MK801+UNC), or MK-801, haloperidol and UNC9994 (MK801+H+UNC). The total expression of Akt (A) corresponds to western blot on Figure 6A; the total expression of Akt (B) corresponds to western blot on Figure 6C. * p < 0.05 in comparison with vehicle-treated animals; N = 3 mice per each experimental group.
